# Innovative Endodontic Management of Pulp Canal Obliteration in Mandibular Incisors Using a Static Navigation System: A Case Report

**DOI:** 10.1002/ccr3.9596

**Published:** 2024-11-20

**Authors:** Reza MahjourianQomi, Mohsen Aminsobhani, Hadi Assadian, Seyed Taha Adnaninia

**Affiliations:** ^1^ Department of Endodontics, School of Dentistry Tehran University of Medical Sciences Tehran Iran; ^2^ Department of Endodontics, Dental Research Center AJA and Tehran University of Medical Sciences Tehran Iran

**Keywords:** 3D‐printed guide, CBCT, mandibular incisors, PCO, static‐guided endodontics

## Abstract

The present case report aims to describe the successful management of pulp canal obliteration (PCO) in mandibular incisors using static‐guided endodontics. A healthy 74‐year‐old man was referred by a prosthodontist for root canal treatment on teeth #31, #32, and #41. Periapical radiographs revealed the presence of periapical lesions as well as PCOs in teeth #31 and #41. After cone‐beam computed tomography (CBCT) evaluation, guided endodontics was selected as the treatment approach. The intraoral scan of the lower jaw and CBCT datasets were aligned and processed through image processing software to design and create a 3D‐printed guide. A virtual drill was superimposed on the scans to ensure proper access to the identified root canals. Finally, the virtually designed guide was printed using a 3D printer and accurately positioned on the teeth. The access cavities were prepared using a Munce Discovery bur. After detecting the canals, the working length was determined. Root canal preparation was performed using rotary files up to F2, followed by obturation with gutta‐percha and a bioceramic sealer. After a six‐month follow‐up, the teeth remained completely asymptomatic and functional, and the periapical radiograph showed a normal periodontal ligament space, demonstrating the effectiveness of the treatment. These results indicate that static‐guided endodontics can be an effective and predictable approach for managing PCO in mandibular incisors with some consideration. Furthermore, these results highlight the clinical implications of this approach, particularly in elderly patients or teeth with severe obliterations.

**Abbreviations**
3D: 3‐dimensional
CAD: computer‐aided design
CBCT: cone‐beam computed tomography
CM: calcific metamorphosis
DICOM: digital imaging and communication
EDTA: ethylenediaminetetraacetic acid
PCC: pulp canal calcification
PCO: pulp canal obliteration
PDL: periodontal ligament
STL: surface tessellation language

**Summary**

- Pulp canal obliteration can result from various causes, including dental trauma, carious and restorations, vital pulp therapy, and physiologic changes.
- Guided endodontics is now used as an alternative method for treating calcified canals, allowing clinicians to reach the root canal space more accurately and conservatively.

## 1 Introduction

Successful treatment in endodontics relies on accessing the root canal and performing chemo‐mechanical preparation of the entire root canal system. Pulp canal obliteration (PCO), also referred to as pulp canal calcification or calcific metamorphosis, is the gradual reduction or complete blockage of the pulp canal space [1, 2]. This is caused by the deposition of hard calcified tissue within the root canal space, leading to pulp atrophy [3]. PCO can result from various causes, including dental trauma, carious and restorations, vital pulp therapy, and physiologic changes in elderly patients [3]. Additionally, orthodontic forces or occlusal overload can induce PCO [2, 3, 4, 5]. Teeth affected by PCO may appear yellow due to reduced tissue transparency and exhibit a diminished or absent response to thermal and electric pulp tests [6]. Preparing the access cavity and locating the canal(s) in teeth with PCO can be difficult, causing complications such as perforation, instrument breakage, and an increased risk of root canal treatment failure [3]. Even experienced endodontists may encounter difficulties in achieving treatment goals when dealing with complete or partial obliteration of the pulp space [2, 3, 7, 8].

Guided endodontics is now used as an alternative method for treating calcified canals, allowing clinicians to reach the pulpal tissue more accurately and conservatively [2]. This approach can prevent unnecessary removal of healthy tooth structure, facilitate root canal location, reduce professional stress, improve patient comfort, enhance precision in planning and treating calcified root canals, and help clinicians overcome challenges in cases of PCOs [1, 8, 9, 10]. Computer‐aided design (CAD) systems are utilized in guided endodontics to merge 3‐dimensional (3D) datasets from cone‐beam computed tomography (CBCT) with tooth surface data obtained from an intraoral scanner. This information is then used to design and create a 3D‐printed stent [11]. This stent enables the drill to access calcified root canals more effectively and minimizes the risk of iatrogenic errors [2, 7, 8, 9, 12]. Previous studies have demonstrated the high accuracy of this method regardless of the operator's experience. Without 3D‐printed guides, the procedure would require multiple sessions and would have a limited prognosis even if performed by an experienced endodontist [8, 13]. This case report aims to describe using a static navigation system for endodontic management of mandibular incisors with PCO.

## 2 Case History/Examination

**FIGURE 1:**
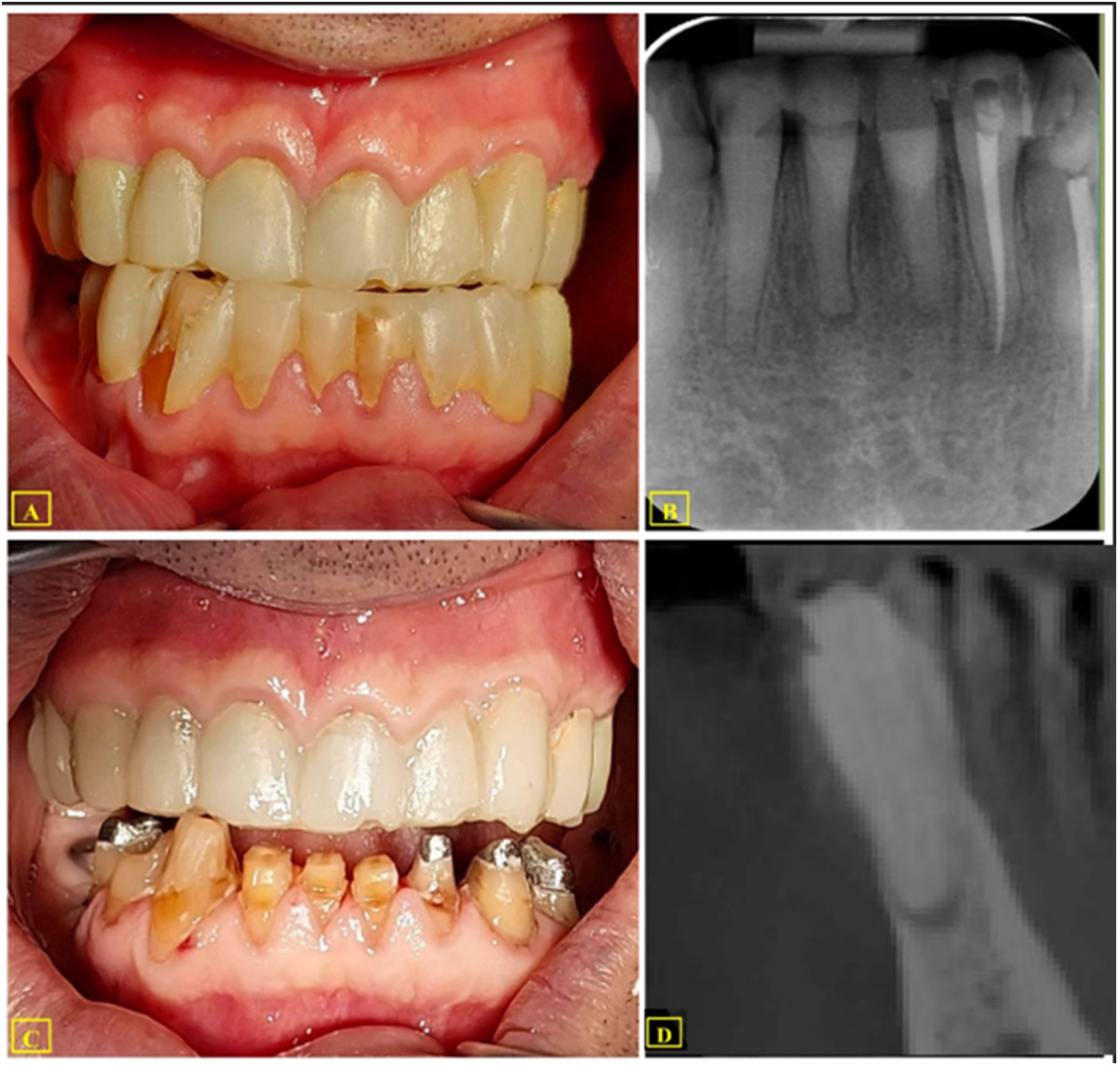
(A) the clinical view; (B) The periapical radiograph (it was 2 months before the start of the root canal treatment); (C) the mock‐up removal and incisal reduction of the teeth; (D) CBCT, Sagital view of tooth #41.

A 74‐year‐old man was referred by a prosthodontist for root canal treatment on teeth #31, #41, and #42 due to the need for full mouth rehabilitation. The patient's medical history was non‐contributory. He was asymptomatic upon clinical examination and reported no pain or discomfort. After removing the mock‐up, the teeth displayed a yellowish discoloration and did not respond to pulp sensibility tests (Figure 1A). There was no sensitivity to percussion or palpation, and the probing depths were within normal limits. No other abnormalities were discovered in hard and soft tissues during the intraoral examination except for the presence of generalized attrition of the incisal surfaces of the teeth and abfractions in cervical areas. Periapical radiographs revealed small periapical lesions in teeth #31 and #41 (Figure 1B). The pulp spaces appeared to be reduced in size and obliterated. All teeth were diagnosed as having necrotic pulps with asymptomatic apical periodontitis in teeth #31 and #41.

## 3 Methods

The occlusal reduction was performed to remove the enamel to facilitate bur penetration, with a reduction of 2 to 3 mm and decrease the pathway of bur penetration to reduce the errors (Figure 1C). Subsequently, a CBCT scan was ordered to further assess the teeth' internal anatomy and external root morphology (Figure 1D). The CBCT scans (ProMax 3D MAX; Planmeca OY, Helsinki, Finland) in sagittal, coronal, and axial view revealed root canal calcification in teeth #31, #41, #42, and periapical lesions in teeth #31, and #41. The root canal pathway was visible between the middle and apical regions in teeth #31 and #41 and between the coronal and middle parts in tooth #42 (Figure 1B).

**FIGURE 2:**
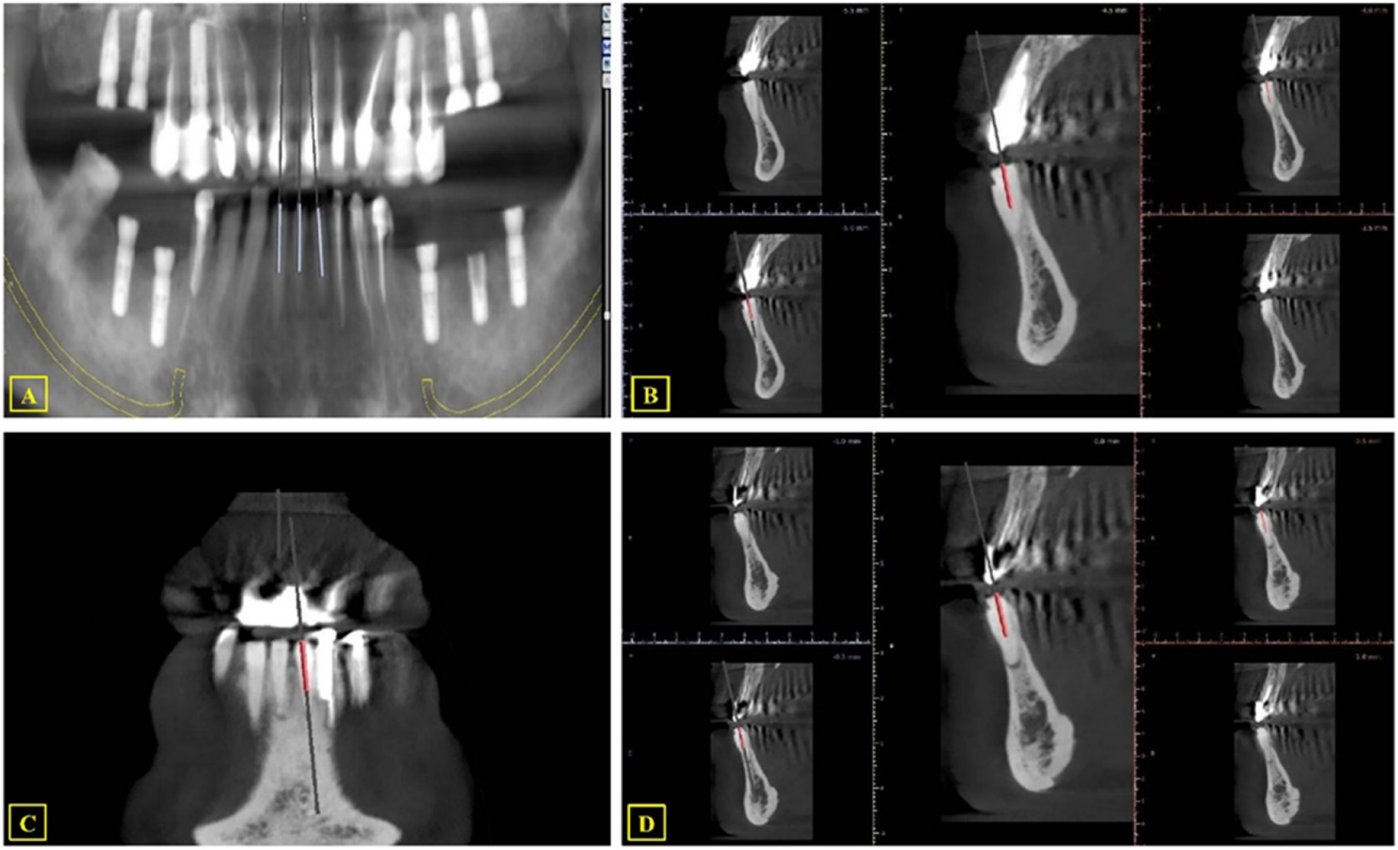
The drill guide path design was based on a three‐dimensional assessment of CBCT slices. (A–D).

After assessing and consulting with the patient, guided endodontics was chosen as the most appropriate treatment approach. The patient provided written informed consent to publish clinical details and images. An intraoral scan (Trios 3 Shape, Warren, NJ, USA) was performed to deliver a faster and more reliable copy of the teeth and soft tissue. CBCT and intraoral image datasets were aligned and processed through image processing software (Dental System v2017; 3Shape, Copenhagen, Denmark). The direction of the canal, the initiation point of the root canal path, and the drilling path were then determined. A virtual drill was superimposed onto the scans to ensure proper access to the identified root canals. The position of the drill was confirmed in three dimensions (Figure 2A–D).

**FIGURE 3:**
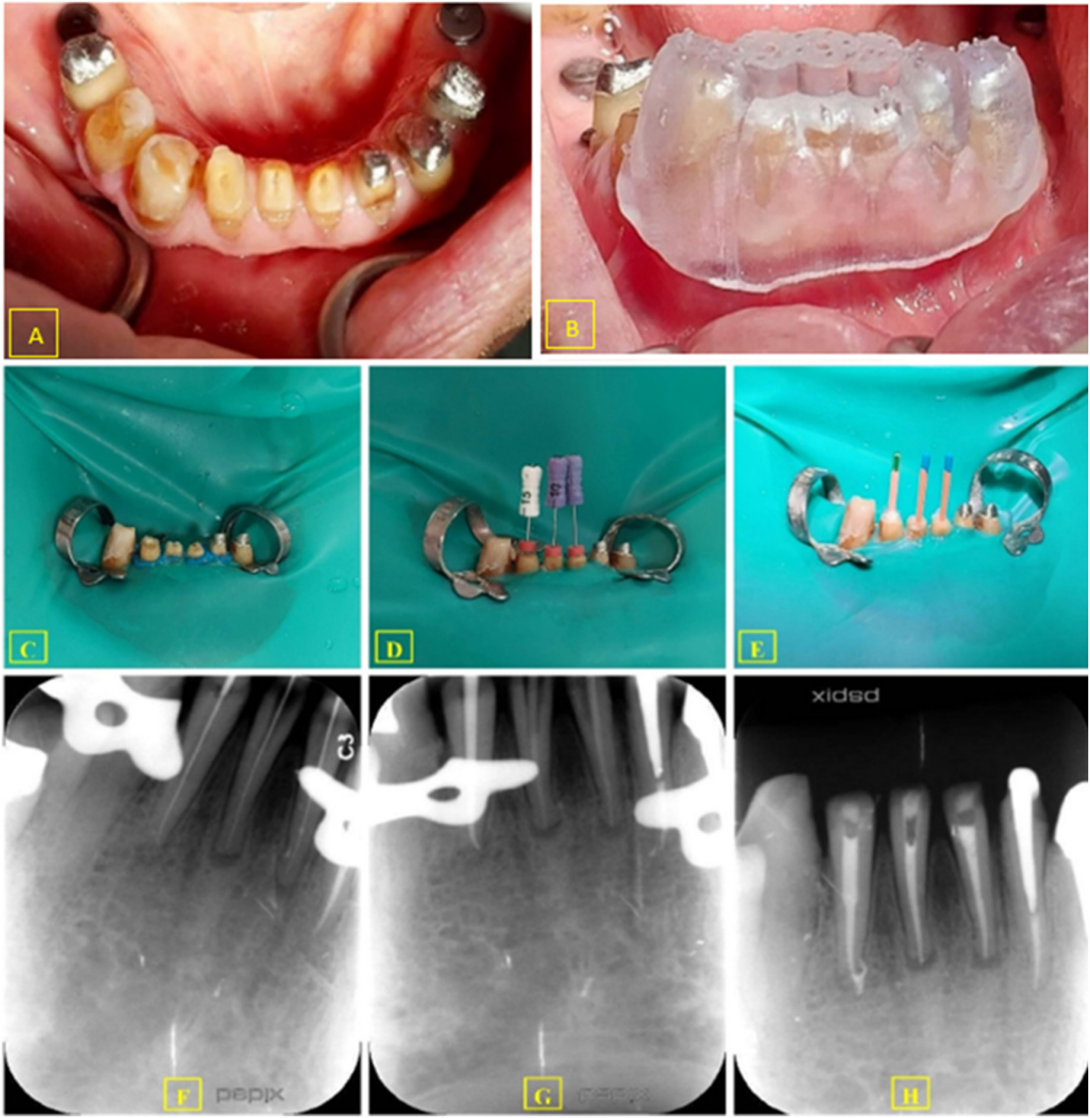
The clinical procedure. (A) The teeth after incisal reduction; (B) checking the correct positioning of the 3D guide; (C) isolation with rubber dam; (D) negotiation of root canals with K‐files; (E, F) determination of the working length; (G) cone fit; (H) post‐obturation radiograph.

During the planning stage, a specific path was created for a size #1 Munce Discovery bur (CJM Engineering, Santa Barbara, CA, USA) by incorporating the bur size into the template. Finally, the virtually designed guide was printed using a 3D printer (Sonic 4 K 3D Printer, Phrozen Technology, Taiwan) and photopolymerized biocompatible polymer resin (PowerResins SG, Singapore). The incisal reduction provided a flat surface for drill insertion (Figure 3A) and the guide was accurately fitted in the correct position (Figure 3B).

The teeth were anesthetized with 1.8 mL of 2% lidocaine and 1/100,000 epinephrine using an buccal infiltration injection. After confirming the fit of the guide, access cavities were prepared through the sleeves using a size #1 Munce Discovery bur (CJM Engineering, Santa Barbara, CA, USA). The drill gradually penetrated, and the position of the drill was checked in each step by radiographs. Initially, teeth #31 and #42 were negotiated, but some challenges occurred with tooth #31, and the periapical radiograph showed some deviation. After ultrasonic troughing under magnification by operating the microscope, the root canal was detected in the correct direction. The teeth were isolated with a rubber dam (Figure 3C). The #10 or #15 K‐files (Micro‐Mega, Besançon, France) were used for root canal negotiation (Figure 3D), and the working length was determined with an electronic apex locator (Endo‐radar plus; Woodpecker Medical Instrument Co. Guilin, China), which was then confirmed radiographically (Figure 3F). The shaping and cleaning were carried out using the S1 to F2 rotary files (Shenzhen Denco Medical Co. Shenzhen, China) with a 5.25% NaOCl irrigation, and apical patency was checked during the instrumentation. A final rinse with 17% ethylenediaminetetraacetic acid (EDTA) (Nikdarman, Tehran, Iran) was performed for 1 minute and then irrigated with normal saline. The root canals were then dried using paper points (Meta Dental Co. Cheougja City, Korea). After taking the cone‐fit radiograph (Figure 3E,G), the obturation was performed using gutta‐percha and a bioceramic sealer (EndoSeal MTA; Maruchi, Wonju, South Korea). The access cavities were temporarily sealed with Cavisol (Golchai, Tehran, Iran), and after final radiograph (Figure 3H), the patient was referred to the prosthodontist to complete the restorative treatment.

## 4 Conclusion and Results

**FIGURE 4:**
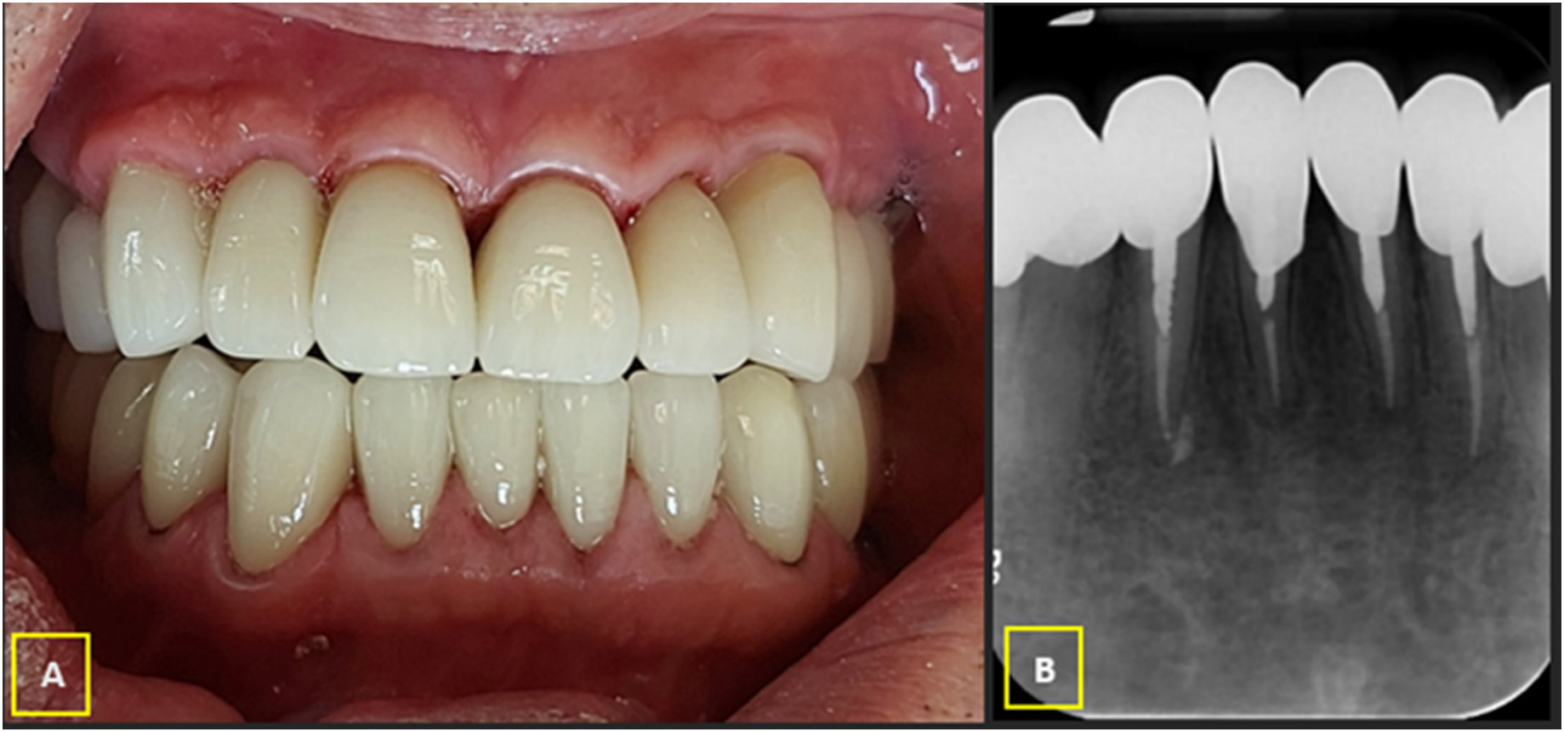
(A) Intraoral photography. (B) Periapical radiograph; At six‐month follow‐up.

After a six‐month follow‐up, the teeth were completely asymptomatic and functional (Figure 4A). The periapical radiograph revealed a normal periodontal ligament (PDL) space around the apex (Figure 4B).

## 5 Discussion

Endodontic management of the calcified root canals poses several challenges in clinical practice. The narrow nature of the canal and the curves in the middle and lower parts of the roots are particularly troublesome, often leading to iatrogenic errors, including perforation and file fracture when attempting to reach working length [2, 7, 8]. Conventional root canal treatment and surgical endodontic treatment are the options in PCO cases; however, the first is time‐consuming, with a risk of excessive removal of tooth structure and prolonged exposure to x‐rays for patients. The latter is also invasive and should be considered when access to the canal cannot be achieved or in cases of severely curved root canals. Currently, guided endodontics is emerging as an effective tool; however, it is primarily used for managing complex cases rather than routine endodontic cases [2, 10, 14].

The present case report details the management of pulp canal obliteration (PCO) in three mandibular incisors (teeth #31, #32, and #41) of a 74‐year‐old man using a static‐guided endodontic approach. A 3D‐printed guide was used to achieve direct access to the root canal system. This approach may require the entry point to be positioned further away from where a clinician typically starts the access cavity preparation. Since the initial report on guided endodontics by Krastl et al. [15], this approach has been widely adopted in several studies. However, treating mandibular incisors presents more significant challenges due to the smaller size of the teeth and the reduced margin for error. Therefore, any mistakes could result in the loss of healthy structure and may lead to iatrogenic errors such as perforations. Despite these challenges, guided endodontics in mandibular incisors is highly effective when utilizing thinner burs [16, 17]. A recent study also demonstrated successful management of mandibular incisors with partial PCOs (teeth #24, #25, and #26) using guided endodontics in a 58‐year‐old man [8].

Guided endodontics offers several benefits over conventional treatments for calcified teeth and is divided into dynamic and static navigation systems. Dynamic‐guided endodontics can be applied to teeth with multiple roots, providing the ability for real‐time adjustment and repositioning, and relies significantly on the clinician's experience [16, 18]. However, static‐guided endodontics is used for straight canals and when sufficient interocclusal space is available. This approach seems expensive and requires a relatively long time to plan, design, and fabricate the 3D printed guides compared to conventional root canal treatment. Static‐guided endodontics is necessary to make reliable copies of the teeth and the CBCT scans that enhance the 3D visualization of the teeth and facilitate identifying and locating obliterated root canals. While digital planning may take longer, patients experience less time on the chair [4, 16, 19]. Radiographical evaluation is crucial in endodontic treatments, from initial diagnosis and treatment planning to evaluating outcomes. Patients are exposed to radiation during guided endodontics, but they receive less radiation compared to conventional treatments. It is highly recommended to use intraoperative radiographs to ensure that the bur is following the correct path without deviation. The tools used in guided endodontics, such as dental instruments, radiography, CBCT, image processing software, and 3D printing, make this approach widely accessible and applicable worldwide [2, 4, 7, 16, 18].

Designing and printing an endodontic guide and its clinical application requires careful attention to each step. The quality of the CBCT acquisition can influence the accuracy of the 3D guide in the digital imaging and communication (DICOM) format and the intraoral scans in the surface tessellation language (STL) format, along with their merging technique using image processing software. In this case, the drill path deviated from the center in tooth 31. This deviation may be due to the unclear and faded depiction of the root canal on the CBCT, combined with an error in the operator's design of the drilling direction. Additionally, the taper of the bur used could have contributed to the deviation. It is recommended to manufacture burs with a parallel shaft and a tip size similar to the bur's diameter to reduce such risks. Therefore, it is essential not to change the position and morphology of the teeth after the CBCT and intraoral scanning processes. According to the manufacturer's recommendations, adjusting printer settings is necessary. The clinician's expertise, the type and dimensions of the drill/bur used for access, and the precise positioning of the guide on the teeth are also crucial in effective treatment [12, 16], whereas guided endodontics is recognized as a highly accurate and effective method for treating PCO [9, 20]. Treating molars with multiple roots, particularly those with tilted or curved roots as revealed on CBCT, requires special consideration. The static‐guided system may have limitations in such cases, as navigating through the complex root canal anatomy can pose a risk of deviation. To minimize complications, careful preoperative assessment is crucial.

In this case report, the long‐term prognosis of static‐guided endodontics was evaluated over a six‐month follow‐up period, during which the treated teeth remained asymptomatic and functional. However, it is essential to consider the potential complications that may arise, particularly in elderly patients who may have altered healing responses. Additionally, teeth with more severe obliterations may present unique challenges during treatment, including difficulties in root canal detection and the risk of procedural errors. Although static‐guided endodontics shows considerable promise, it is crucial to approach these potential complications with care to ensure successful outcomes. Further clinical studies involving a larger number of patients with sufficient follow‐up periods are needed to evaluate the efficacy of this technique more thoroughly. Nevertheless, future studies should compare the effectiveness of different planning software, materials, and designs for 3D guides and burs.

## Author Contributions

**Reza MahjourianQomi:** conceptualization, data curation, investigation, methodology, project administration, writing – original draft, writing – review and editing. **Mohsen Aminsobhani:** data curation, investigation, supervision, writing – original draft, writing – review and editing. **Hadi Assadian:** conceptualization, supervision, visualization, writing – original draft. **Seyed Taha Adnaninia:** investigation, project administration, writing – original draft, writing – review and editing.

## Consent

Written informed consent was obtained from the patient to publish this report following the journal's patient consent policy.

## Conflicts of Interest

The authors declare no conflicts of interest.

## Data Availability

The data that support the findings of this study are available on request from the corresponding author.
